# Function of cone and cone-related pathways in Ca_V_1.4 IT mice

**DOI:** 10.1038/s41598-021-82210-7

**Published:** 2021-02-01

**Authors:** Lucia Zanetti, Irem Kilicarslan, Michael Netzer, Norbert Babai, Hartwig Seitter, Alexandra Koschak

**Affiliations:** 1 Institute of Pharmacy, Pharmacology and Toxicology, Center for Chemistry and Biomedicine, University of Innsbruck, Innrain 80-82, 6020 Innsbruck, Austria; 2 Department of Biology, University of Erlangen, 91058 Erlangen, Germany

**Keywords:** Neuroscience, Retinopathy of prematurity

## Abstract

Ca_V_1.4 L-type calcium channels are predominantly expressed in photoreceptor terminals playing a crucial role for synaptic transmission and, consequently, for vision. Human mutations in the encoding gene are associated with congenital stationary night blindness type-2. Besides rod-driven scotopic vision also cone-driven photopic responses are severely affected in patients. The present study therefore examined functional and morphological changes in cones and cone-related pathways in mice carrying the Ca_V_1.4 gain-of function mutation I756T (Ca_V_1.4-IT) using multielectrode array, patch-clamp and immunohistochemical analyses. Ca_V_1.4-IT ganglion cell responses to photopic stimuli were seen only in a small fraction of cells indicative of a major impairment in the cone pathway. Though cone photoreceptors underwent morphological rearrangements, they retained their ability to release glutamate. Our functional data suggested a postsynaptic cone bipolar cell defect, supported by the fact that the majority of cone bipolar cells showed sprouting, while horizontal cells maintained contacts with cones and cone-to-horizontal cell input was preserved. Furthermore a reduction of basal Ca^2+^ influx by a calcium channel blocker was not sufficient to rescue synaptic transmission deficits caused by the Ca_V_1.4-IT mutation. Long term treatments with low-dose Ca^2+^ channel blockers might however be beneficial reducing Ca^2+^ toxicity without major effects on ganglion cells responses.

## Introduction

Ca_V_1.4 L-type calcium channels (LTCCs) are key players in retinal photoreceptor ribbon synapses^1–5^ sustaining continuous vesicle release during prolonged depolarization^6,7^. The importance of Ca_V_1.4 LTCCs is testified by numerous mutations in the *CACNA1F* gene which encodes for the pore-forming Ca_V_1.4 α1 subunit that lead to a retinal disease known as congenital stationary night blindness type 2 (CSNB2; for review see Zeitz et al.^8^). CSNB2 symptoms include low visual acuity, strabismus, nystagmus and photophobia; night blindness and myopia can be present or not. Despite this phenotypic variability, not only due to the broad spectrum of mutations^9,10^, CSNB2 is diagnosed by the electroretinogram (ERG). CSNB2 patients show a normal a-wave but typically a reduced b-wave, in both rod- (scotopic) and cone-driven (photopic) ERGs. This phenotype is consistent with a dysfunctional transmission from photoreceptors to bipolar cells^8,11^.

Previous studies highlighted the importance of Cav1.4 channels for the assembly and maintenance of photoreceptor ribbon synapses in mice^12–14^ and fish^15^. In rod photoreceptors of different CSNB2 mouse models, dysregulation of the channel led not only to changes in the ribbon structure but also to axonal retraction^16–18^ and eventually to rod bipolar and horizontal cell dendritic sprouting^4,16,18–25^. Ectopic synapses between rods and sprouting second order neurons have been found in the outer nuclear layer (ONL) of different CSNB2 animal models^16,18,20,26^. But so far only in the G305X Ca_V_1.4 knockout (KO) mouse model ectopic synapses between cones and rod bipolar cells in the ONL were reported, along with progressive degeneration and structural abnormalities of cone photoreceptor^27^. However, all the Ca_V_1.4 KO models published so far showed functional features that were more severe than in humans carrying loss-of-function mutations. Knoflach et al. could not detect any light triggered ganglion cell responses in a Ca_V_1.4 KO model^22^, while Mansergh et al., reported that the premature truncation of channels leads to functionally blind mice without detectable responses in the visual cortex^19^.

In this study we investigated a mouse model carrying a single point mutation in Ca_V_1.4 that leads to the substitution of an isoleucine with a threonine in position 745 in the human Ca_V_1.4 protein (Ca_V_1.4-I745T). First identified in a New Zealand family, this mutation causes a severe CSNB2 phenotype accompanied by cases of intellectual disabilities within the male members of the family^28,29^. In heterologous expression systems, Ca_V_1.4-IT channels showed a gain-of-function phenotype^29^. The mouse model carrying the corresponding mutation (Ca_V_1.4-I756T) was previously validated as a proper model to study the CSNB2 phenotype^4,21,23^. The ERGs showed rod- and cone-driven a-waves, but a reduction of the scotopic and photopic b-waves reflecting ON and OFF bipolar cell function in Ca_V_1.4-IT mice comparable to human ERGs ^4,21,23^. While Ca_V_1.4 is not only expressed in rod and cone photoreceptors but has also been shown in bipolar cells its particular role and contribution to cellular Ca^2+^ influx there is still elusive^1,19,30^. While sprouting of second order rod bipolar and horizontal cells has been found in Ca_V_1.4-IT retinas^4,21,23^, and literature previously focused on rod pathway connections morphological data about cone bipolar cells in CSNB2 models are scarce^25,31^ and the degree of synaptic remodelling is unknown.

Therefore we focused on cone signalling pathways of Ca_V_1.4-IT retinas. Measuring light induced ganglion cell activity while isolating specific signalling pathways (through different light levels as well as pharmacologically) we showed that the cone pathway was severely affected albeit the tonic vesicle release from cones was comparable to wild type. This was remarkable because our immunohistochemical analyses revealed that cone pedicles lost their regular mosaic arrangement in the outer plexiform layer (OPL) and cone bipolar cell dendrites extended into the ONL. Because the rod pathway was still responsive in Ca_V_1.4-IT retinas we also tested whether the functional changes seen in synaptic transmission could be reversed by L-type calcium channel specific drugs.

## Materials and methods

### Animals

Animals were housed in groups of 2–6 per cage under standard laboratory conditions (12:12 light/dark, lights on at 07:00 h, 22 ± 2 °C, 50–60% humidity) with food and water available ad libitum. Experimental procedures were designed to minimize animal suffering and the number of used animals and approved by the national ethical committee on animal care and use (Austrian Federal Ministry for Science and Research). All methods were performed in accordance with the relevant guidelines and regulations.

### Ca_V_1.4 mouse lines

We used two mouse models, Ca_V_1.4-IT and Ca_V_1.4-KO (Cacna1fΔEx14-17), previously described in^21,22^. Both male and female mice were investigated. Genotyping was performed as described in^21^.

### Immunohistochemistry

10 to 15 weeks old mice were anesthetized with isoflurane (Vetflurane®k, Virbac) and killed by cervical dislocation. *Vertical sections:* The following steps were conducted at room temperature if not stated otherwise. Eyes were quickly removed from the eye socket, opened at the scleral-corneal rim and immersed for 10 min with 4% paraformaldehyde (PFA) in 1X phosphate-buffered saline (1X PBS, pH 7.4). Cornea, lens and vitreous were removed. Eye cups were then fixed with 4%PFA/1X PBS for 20 min, washed four times with 1X PBS and cryoprotected by a graded sucrose series: 10% sucrose in 1X PBS for 1 h, 20% sucrose in 1X PBS for 1 h and 30% sucrose in 1X PBS overnight at 4 °C. Eyecups were orientated along the dorsoventral axis, embedded in OCT Medium (Tissue-Tek O.C.T Compound; Sakura Finetek, Tokyo, Japan) and frozen in liquid nitrogen. Vertical sections (16 µm) were cut on a cryostat (Leica Microsystems, Wetzlar, Germany), mounted on gelatine coated slides and stored at − 20 °C. For immunofluorescence experiments, sections were washed three times in 1X PBS-T (1X PBS + 0.1% Triton X-100, Sigma-Aldrich, St. Louis, MO, USA), blocked for 1 h in 1X PBS-T containing 1% bovine serum albumin (BSA, Sigma-Aldrich, A7030) and incubated overnight at 4 °C with primary antibodies diluted in 1X PBS-T at concentrations listed in Supplementary table 1. After washing three times with 1X PBS-T, sections were incubated with the secondary antibodies (Supplementary table 2) for one. Additional washes preceded the counterstaining with DAPI (1:10,000; Sigma, D-9542) and eventually the sections were mounted using Poly/Mount (Polysciences, Inc., Warrington, PA, USA).

For whole-mounts eyecups were fixed for 30 min in 4% PFA/1X PBS. The retina was dissected by removing the sclera and incubated in blocking solution (1% BSA in 1X PBS-T with 0.02% sodium azide) for 1 h. Primary antibodies were diluted in antibody solution (1% BSA in 1X PBS + 1% Triton X-100 with 0.02% sodium azide) and incubated for 1 week on a shaker. Afterwards, whole-mounts were washed three times in 1X PBS for 30 min. Secondary antibodies were diluted in 1X PBS-T with 0.02% sodium azide and incubated overnight at room temperature on a shaker. After washing again three times in 1X PBS-T for 30 min, whole-mounts were flattened by cutting 4 times (“clover-leaf” cuts) and mounted using Aqua Poly/Mount (Polysciences, Inc.).

Sections and whole-mounts were imaged with a confocal laser scanning microscope (Leica TCS SP5-II; Leica Microsystems, Wetzlar, Germany) at 40× magnification (NA 1.30). Series of micrographs were taken at 0.25 and 0.42 µm intervals and collapsed to a z-projection with maximum intensities in ImageJ (National Institutes of Health, Bethesda, Maryland, USA). The analysis of retinal layer thickness, soma and pedicle size and the quantification of cell numbers was conducted using ImageJ. Images were adjusted for contrast, brightness using ImageJ and assembled in Adobe Photoshop CS5.

### Microelectrode array recordings

Mice were dark-adapted for at least 2 h before the experiment, and sacrificed by cervical dislocation after isoflurane anaesthesia between circadian zeitgeber time ZT5.5 and ZT8.5. Animals were 10 to 15 weeks old at the time of the experiment. The ventral position on each eye was marked with a soldering tool (BP645CEU 6W, Weller, Apex, NC, USA) before excision, and the eyes were put in bath solution (in [mM]: 110 NaCl, 2.5 KCl, 1 CaCl_2_, 1.6 MgCl_2_, 10 D-Glucose, and 22 NaHCO_3_; bubbled with 5% CO_2_/95% O_2_) for dissection. After cornea and lens removal, the retina was isolated and mounted on a dark grey nitrocellulose filter (13006-50-ACN, Sartorius Stedim, Göttingen, Germany) with a central 3 × 3 mm aperture, with the dorsal part of the retina placed as centred as possible. All operations were performed under dim red light conditions.

Recordings were carried out with perforated 120-electrode micro-electrode arrays (MEA; 120pMEA100/30iR-Ti-pr, Multichannel Systems, Reutlingen, Germany). Experiments were performed as described^32^. Briefly, the dorsal retina was placed ganglion cell-side down in the recording chamber and continuously perfused with fresh bath solution at 30 °C. Dorsal part was chosen based on cone opsin spectral distribution and visual stimulation spectra, see below. Raw data were recorded at 25 kHz with a MEA-system (MEA2100, Multichannel Systems, Reutlingen, Germany).

Light stimulation was performed as described^32^. Briefly, a computer-controlled digital light processing projector (Lightcrafter E4500MKII, EKB Technologies Ltd, Bat Yam, Israel) was used to stimulate the retina with greyscale visual stimuli. We used the built-in blue and green LED matching well the Rhodopsin and M opsin spectra. The light path was integrated with two sets of neutral density (ND) filters (63–390, 63–393, 63–395, Edmund Optics, York, UK) that allowed us to set scotopic (ND8) and eventually photopic (ND4) light stimuli. We presented the same set of visual stimuli at each ND-level during an experiment. Full-field flashes consisted of 1-s negative and positive contrast steps (50% Weber contrast) with 5-s of background grey (grey value 200) in between. We analysed the maximum positive responses after either the bright or the dark flash.

We blocked glutamate metabotropic mGluR6 receptors by adding L-2-amino-4-phosphonobutyric acid (L-AP4, 20 µM, Tocris, Cat. No. 0103) or glycinergic receptors by adding 2 µM strychnine (Sigma Aldrich, Cat. No. S8753) to the bath solution. Nilvadipine (catalog #5711, Tocris Bioscience) was used to block LTCCs. Drugs were added to the bath solution, and drug perfusion started at least 10 min prior to the recording.

Retinal spikes were extracted from high-passed filtered (500 Hz, 10th-order butterworth filter) traces using Matlab (The Mathworks Inc., MA, USA). Spike sorting was carried out as described in^33^, using a custom-made Matlab script. The latency of response was defined as described^21^.

### Whole-cell patch clamp recordings from horizontal cells

#### Slice preparation

Horizontal slice preparation was performed as described^34^. Briefly, the retina was cut in four pieces which were embedded into 1.8% low-melting agarose dissolved in Ames´ Medium (US Biological, Salem, MA). Approximately 150 µm thick slices were cut with a vibratome (Leica Microsystems, Wetzlar, Germany) at room light condition. Slice were kept in Ames´ Medium at 37 °C (pH 7.2) in an incubator containing 5% CO_2_ and 55% O_2_. Whole-cell recordings were carried at room temperature. 63X water immersion objective (Zeiss, Jena, Germany) was mounted on a fixed-stage microscope (Zeiss Axio Examiner) equipped with Dodt contrast. Currents were recorded with an EPC-10 patch-clamp amplifier (Heka Elektronk, Lambrecht, Germany) low‐pass filtered at 2.9 kHz using a built‐in Bessel filter, and digitized at 10 kHz with Patchmaster software (Heka Elektronik). Patch pipettes were pulled from borosilicate glass (Sutter Instruments, Novato, CA, USA) to a final resistance of 4‐7 MΩ. Series resistance was compensated up to 50%.The extracellular solution contained (in [mM]): 125 NaCl, 25 NaHCO_3_, 1.25 NaH_2_PO_4_, 2.5 KCl, 2 CaCl_2_, 1 MgCl_2_, 10 glucose, 1 ascorbic acid, 2 Na-pyruvate (pH 7.4). Intracellular solution contained (in [mM]): 134.5 K‐gluconate, 10 KCl, 5 EGTA, 10 HEPES, 1 CaCl_2_, 1 MgCl_2_, 4 Mg‐ATP, 0.5 GTP (pH 7.2). Analysis of tonic postsynaptic current was carried out with the Clampfit 10.7 (Molecular Devices, CA, USA).

### Statistical analysis

Data are presented as mean ± SEM, unless stated otherwise for the indicated number of experiments or cells analysed (n) from the indicated number of animals (N). Data analysis was performed using Clampfit 10.2 (Axon Instruments), Matlab (The Mathworks Inc., MA, USA, GraphPad Prism 5 (GraphPad Software). D´Agostino and Pearson omnibus normality test was used to detect deviations from normality. Means per animal were considered normally distributed if the single data points showed a normal distribution. Data were analysed by unpaired Students *t* test, Mann–Whitney *U* test, one-way ANOVA with Bonferroni post hoc test and Kruskal–Wallis with Dunn’s multiple comparison post hoc test as appropriate and indicated for individual experiments. Statistical significance was set at *p* < 0.05. Significance levels of *p* < 0.05, < 0.01, or < 0.001 are denoted in graphs by a single, double, or triple asterisk, respectively.

## Results

In this study we provide further insight in rod and cone synaptic dysfunction in a mouse line that harbours the Ca_V_1.4 gain-of-function mutation Ile-to-Thr at residue 756 (Ca_V_1.4-IT;^4,21–23^) reported in human patients^28,29^. Because also photopic responses are severely affected in patients with CSNB2^8,35^, yet previous analyses emphasized on the rod system, we focused primarily on the cone pathway. To this end, we characterized rod and cone pathways at the ganglion cell output using multielectrode array (MEA) recordings and correlated them with morphological analyses.

### Ca_V_1.4-IT ganglion cell responses are more affected under photopic light stimulation

**Figure 1:**
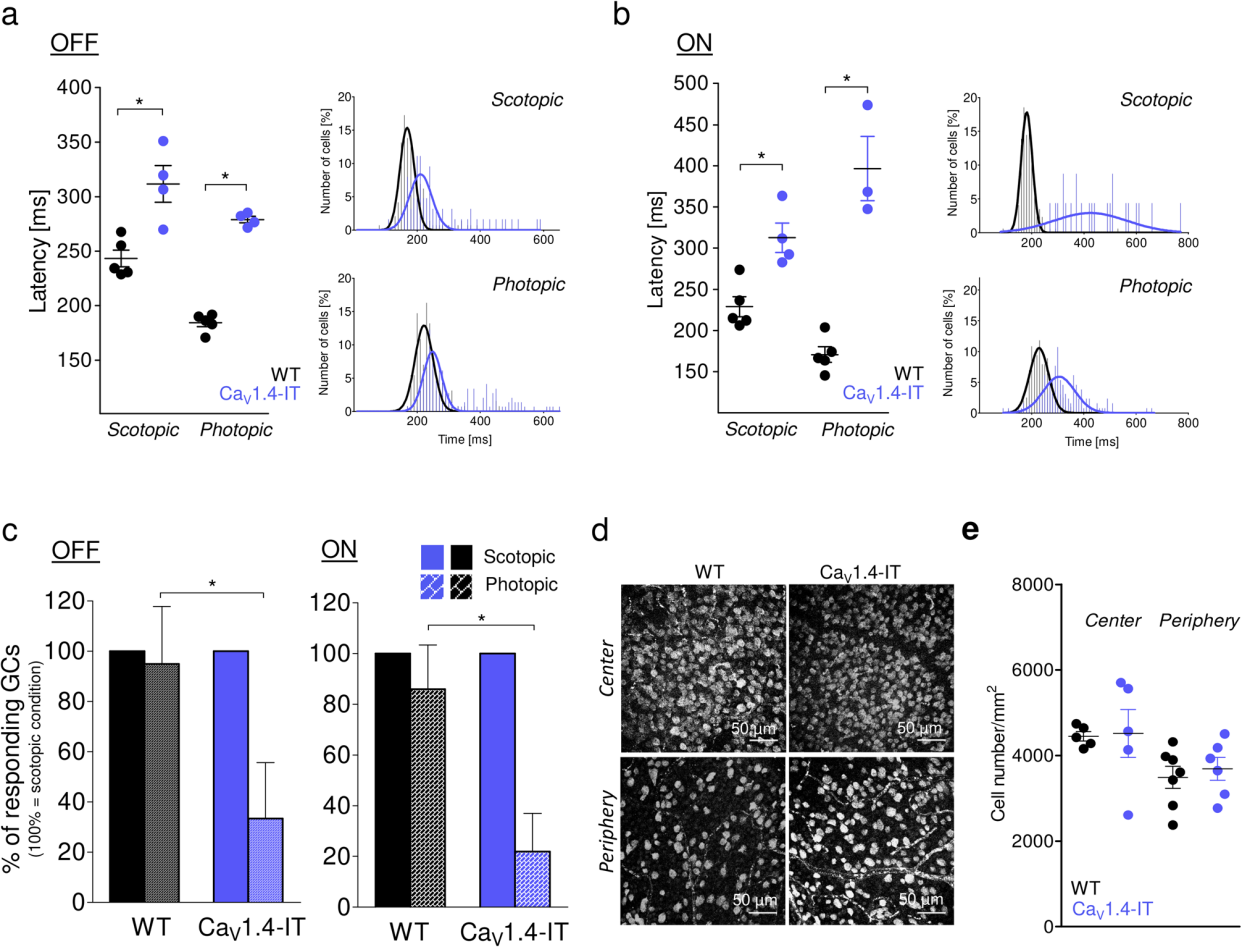
Light induced ganglion cell activity in wild type and Ca_V_1.4-IT retinas. A 1 s dark or bright full field flash was used to assess ganglion cell OFF and ON light responses in wild type (WT, black) and Ca_V_1.4-IT (blue) retinas. (**a**) Left panel: mean latencies of OFF ganglion cells under scotopic and photopic conditions in WT ([ms]: scotopic: 243.3 ± 7.7; photopic: 184.4 ± 3.7, N = 5) and Ca_V_1.4-IT ([ms]: scotopic: 311.8 ± 16.8; photopic: 279.0 ± 3.0, N = 4). Statistics: *, *p* = 0.0159, two-tailed Mann Whitney test. Right panel: histogram of the OFF ganglion cell response delay. (**b**) Left panel: mean latencies of ON ganglion cell responses in WT ([ms]: scotopic: 228.9 ± 12.4; photopic: 170.8 ± 9.5, N = 5) and Ca_V_1.4-IT ([ms]: scotopic: 312.8 ± 18.02; photopic: 396.7 ± 39.08, N = 4). Statistics: scotopic: *, *p* = 0.0159, photopic: *, *p* = 0.0357, two-tailed Mann Whitney test. Right panel: histogram of the ON ganglion cell response delay. Bin width: 10 ms. WT: N = 5, Ca_V_1.4-IT N = 4. In (**c**), the ganglion cell number responding upon photopic stimulation was normalized to the number of cells responding under scotopic conditions. Note that the percentage of OFF and ON ganglion cells that responded upon photopic stimulation after the scotopic light flash was significantly reduced in Ca_V_1.4-IT mice. WT: N = 5, Ca_V_1.4-IT: N = 4, Statistics: OFF ls: *, *p* = 0.0159, ON: *, *p* = 0.0357, two-tailed Mann Whitney test. (**d**) Representative example of ganglion cells positive for RNA-binding protein with multiple splicing. (**e**) The number of stained cells per 1mm^2^ was compared: WT: 4450.6 ± 108.9 and 3491.9 ± 257.8; Ca_V_1.4-IT: 4516.1 ± 560.8 and 3688.6 ± 268.1 in center and periphery, correspondingly; WT: N = 7, Ca_V_1.4-IT: N = 6. All data are presented as mean ± SEM.

First, we dissected ganglion cell responses triggered by rod and cone activation using scotopic and photopic full-field flash stimuli in MEA recordings. We subdivide them into OFF and ON ganglion cells using dark and bright flashes (Fig. 1). OFF ganglion cells increase their firing rate upon dark flashes (negative contrast, Fig. 1a), while ON ganglion cells increase firing rate to bright flashes (positive contrast, Fig. 1b). We found a higher baseline firing frequency under both light conditions ([Hz]: scotopic: WT = 1.2 ± 0.1, Ca_V_1.4-IT = 2.2 ± 0.2 ***; photopic: WT: 1.6 ± 0.12, Ca_V_1.4-IT: 3.2 ± 0.2 ***p < 0.01; WT: N = 5, Ca_V_1.4-IT: N = 4; mean ± SEM; Statistics: ***p < 0.01, Kruskal–Wallis test with Dunn's multiple comparison test; for mesopic light see Knoflach et al.^22^) together with a significantly increased latency in OFF and ON ganglion cell responses (Fig. 1a,b; compare mesopic light: Knoflach et al.^22^). More strikingly, in the Ca_V_1.4-IT retina less than one third of the ganglion cells responding to scotopic stimulation also showed a photopic response (21.9% of ON and 33.4% of OFF ganglion cells), whereas in wild type the majority of ganglion cells responded under both light conditions (Fig. 1c). Immunohistochemical analyses with the ganglion cell marker RNA-binding protein with multiple splicing showed comparable ganglion cell numbers in central and peripheral wild type and Ca_V_1.4-IT retinas (Fig. 1d,e). Our data indicated that rod-driven responses are largely preserved whereas cone-driven pathways are strongly impaired. The impairment in the cone pathway could either be explained by a defect at the cone-to-cone bipolar cell synapse or further downstream at the cone bipolar-to-ganglion cell synapse.

Because rod pathways require cone bipolar cells to contact ganglion cells (for pathways see supplementary Fig. 2: primary rod → ON rod bipolar cell → AII amacrine cell → cone bipolar cell → ganglion cell; secondary rod U → cone → cone bipolar cell → ganglion cell), a functional rod pathway also indicates functional cone bipolar-to-ganglion cell synapses. Under scotopic illumination the number of responding ganglion cells was similar in wild type and Ca_V_1.4-IT mice (number of cells per experiment: WT: 67.6 ± 4.7, N = 5; Ca_V_1.4-IT: 64.5 ± 8.9; N = 4; mean ± SEM) suggesting that ganglion cells still received input from cone bipolar cells. Given that, we further focused on the first synapse, between cones and cone bipolar cells.

**Figure 2:**
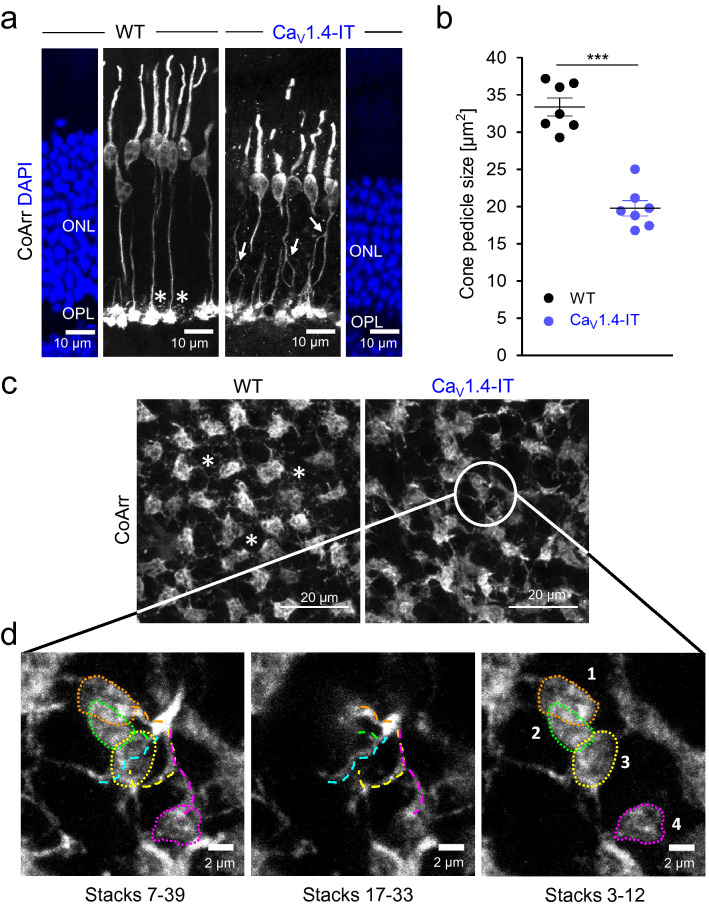
Cone photoreceptor terminals in Ca_V_1.4 mutant retinas. (**a**) Cone photoreceptors were positive for cone arrestin (CoArr). Their terminals are located in the outer plexiform layer (OPL) as can be derived from the DAPI counterstainings (blue) of the vertical sections. Asterisks indicate telodendrial processes. Arrows point to axonal branches in the ONL. (**b**) The size of the cone pedicles was measured in whole mount stainings of WT (black) and Ca_V_1.4-IT (blue) retinas: [µm^2^]: WT: 33.5 ± 1.2; Ca_V_1.4-IT: 19.9 ± 1.3. WT: N = 7, n = 74; Ca_V_1.4-IT: N = 7, n = 78. ***, *p* = 0.0006, Mann Whitney U test. Data are presented as mean ± SEM. (**c**) Retinal whole-mounts were stained with CoArr to reveal cone terminals. Asterisks indicate telodendrial processes. In Ca_V_1.4-IT retinas, the circle defines a cluster of small sized cone pedicles. Representative pictures are shown. (**d**) Several branches of a cone axon were tracked and are shown in different planes indicated by the stack numbers. The rightmost panel shows that four small pedicles of one cone form the cluster seen in (**c**). Colours were used to better visualize axonal sprouting and the different cone pedicles. WT: N = 7; Ca_V_1.4-IT: N = 7.

### Morphology of cone photoreceptor synaptic terminals in Ca_V_1.4-IT retinas

We and others previously reported a reduction in the size of the ONL in the Ca_V_1.4-IT retinas^21,23^ indicating a loss of (rod) photoreceptors. To now also quantify the number of cones, we stained whole-mount retinas with an anti-cone arrestin antibody. The number of cones was comparable in wild type and Ca_V_1.4-IT (wild type: 458.8 ± 43.6, Ca_V_1.4-IT: 431.5 ± 32.0; mean per 0.0375 mm^2^: ± SD; Statistics: Mann Whitney U-test: *p* = 0.18, numbers extrapolate to 12,235 and 11,506 cells/mm^2^, in line with wild type cone densities^36^) whereas the total number of photoreceptors was reduced (counted in DAPI-stained sections: wild type: 542.6 ± 28.7, Ca_V_1.4-IT: 409.6 ± 39.5, mean per 193.75 µm linear retinal length ± SD; Statistics: Mann Whitney U-test: *p* = 0.0003). Thus, cone photoreceptors do not undergo significant cell death in Ca_V_1.4-IT retinas within the age tested here.

In previous analyses Ca_V_1.4-IT cone terminals seemed enlarged^22^. Re-visiting cone terminal morphology revealed that what seemed like enlarged pedicles was a cluster of cone terminals and individual pedicles were significantly smaller than wild type (Fig. 2a,c,d). We tracked cone axons through different layers of a z-stack and observed clusters of pedicles which either derived from a single or different cones (Fig. 2d), displaying axonal branching. The branches occurred in different levels of the ONL (see Fig. 2a, arrows). In the central retina of a few mice (1 wild type and 2 Ca_V_1.4-IT) we were able to count cone somata (in the ONL) and pedicles (in the OPL) in the same scanned image. While in the wild type the pedicles to soma ratio was almost 1:1 (only ~ 2% more pedicles than somas), we found 45% more synaptic terminals than cone somas in Ca_V_1.4-IT, suggesting that axonal branching is a common feature.

Cones couple to surrounding photoreceptors at the tip of fine processes, so-called telodendria^37–39^ which emerge from their pedicles. Our cone arrestin staining suggested irregular telodendrial contacts between photoreceptors (Fig. 2a,c) in Ca_V_1.4-IT retinas which might be due to rod terminal retraction (Supplementary Fig. 1,^4,22,23^). Together our analyses elicited that cones changed more profoundly than previously appreciated, however retained their synaptic terminals in the OPL (Fig. 2a). We therefore set out to investigate their synaptic function.

### Functionality of the cone pathway in Ca_V_1.4-IT retinas

**Figure 3:**
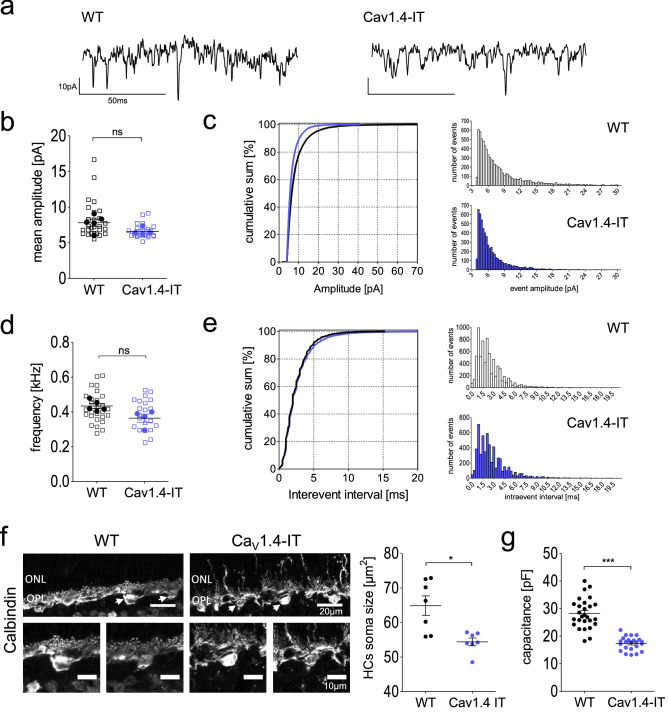
Horizontal cell activity as a readout of cone function. (**a**) Representative traces recorded from horizontal cell body of a wild type (WT, left) retina and Ca_V_1.4-IT (right), 120 ms displayed. In (**b**), the mean amplitude [pA] is indicated for: WT: 7.8 ± 0.5, Ca_V_1.4-IT: 6.64 ± 0.3. No statistical difference was found in a two-tailed Mann Whitney U-test. WT: N = 5; Ca_V_1.4-IT: N = 4. (**c**) Left panel: cumulative sum of EPSC amplitudes recorded in WT and Ca_V_1.4-IT horizontal cells; Statistics: ***, *p* < 0.001, two sample Kolmogorov–Smirnov test. Right panel: histogram of the amplitude distribution of WT (top, 7504 events from 24 cells) and Ca_V_1.4-IT (bottom, 5653 events from 22 cells). Bin width, 0.3 pA. In (**d**) the mean frequency of the events recorded in WT (434.6 ± 13.9 Hz) and Ca_V_1.4-IT (392.5 ± 11.1 Hz) showed no statistical difference in a two-tailed unpaired t-test. WT: N = 5; Ca_V_1.4-IT: N = 4. (**e**) Left panel: cumulative sum of EPSC the intra-event interval. Right panels: histogram of the intra-event interval of WT (top, 7504 events from 24 cells) and Ca_V_1.4-IT (bottom, 5653 events from 22 cells). Bin width, 0.3 ms. In (**b**) and (**d**), empty squares in the dot plot represent the average of 300 events from the same trace, the statistical analysis was, however, carried out for the means per animal (full circles). **(f)** Immunostaining of horizontal cells (calbindin) in WT and Ca_V_1.4-IT retinas. Arrows mark the somas of the horizontal cells shown in the lower panels. (**g**) Left panel: horizontal cell soma size measurement from WT and Ca_V_1.4-IT retinal sections also showed a reduction in the mean horizontal cell soma size [µm^2^]: WT: 65.1 ± 2.2, N = 7, n = 47; Ca_V_1.4-IT: 54 ± 1.5, N = 7, n = 42. Statistics: *, *p* = 0.0175, Mann Whitney U test. Right panel: extraction of whole-cell capacitance values (mean [pF] for WT: 28.2 ± 1.04; N = 27 and Ca_V_1.4-IT: 17.4 ± 0.5, N = 23) indicated a reduction in horizontal cell size in Ca_V_1.4-IT compared to wild type retinas. Statistics: ***, *p* < 0.0001, two-tail unpaired t-test. All data are presented as mean ± SEM.

To explore the glutamate release from cones, we performed whole-cell patch-clamp recordings in postsynaptic horizontal cells as a read-out for transmitter release from cone presynaptic terminals. In horizontal cells the constant release of glutamate from cone photoreceptors results in a persistent inward current accompanied by tonic activity composed of high-frequency excitatory postsynaptic currents (EPSCs, Fig. 3a, see also Feigenspan & Babai^40^). Under laboratory light conditions, the cumulative distribution of Ca_V_1.4-IT EPSC amplitudes was significantly shifted to the left (Fig. 3c), indicating more events with smaller current amplitude. However, we saw no difference in the mean amplitude of the EPSCs (Fig. 3b) between wild type and mutant mice. Moreover, neither the inter-event interval nor the overall event frequency showed any statistical difference (Fig. 3d,e). Ca_V_1.4-IT cones therefore released glutamate at a comparable rate as in wild type meaning the cone terminal per se is functional. Interestingly, we found a reduction in cell membrane capacitance as a measure for horizontal cell size (Fig. 3g). This finding is in good agreement with our morphological data which showed a reduction in the soma size of calbindin-stained horizontal cells in Ca_V_1.4-IT retinas compared to wild type (Fig. 3f).

**Figure 4:**
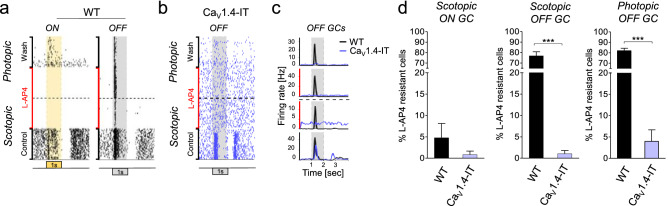
Defective OFF signalling pathways in Ca_V_1.4-IT retinas. OFF pathways were pharmacologically isolated by application of 50 μm L-AP4 (red). Ganglion cell spiking activity was recorded during scotopic and photopic illumination (separated by the horizontal dashed line), in the absence (control, wash) or presence of L-AP4 (red). For each condition 40 repetitions of the same stimulus are shown. (**a**) Representative examples of ON ganglion cell activity upon a positive contrast flash (yellow) and OFF ganglion cell activity upon negative contrast flash (grey). Note that in the presence of L-AP4, Ca_V_1.4-IT OFF ganglion cells did not anymore respond to light (**b**). Panel (**c**) show the superimposed peri-stimulus time histogram of the OFF ganglion cells shown in (**a**,**b**): from bottom to top: scotopic—control, scotopic—L-AP4, photopic—L-AP4, and wash out (wash).(**d**) Percentage of ganglion cells responding under L-AP4 perfusion upon different light levels. Of note, no responding ON ganglion cells were detected under photopic conditions. The number of responding cells prior to perfusion with L-AP4 (WT, ON n = 70; OFF n = 78; Ca_V_1.4-IT, ON n = 91; OFF n = 189) was set to 100%. Percentage of responding ganglion cells under L-AP4 perfusion (% L-AP4 resistant cells): scotopic: WT: OFF: 76.9 ± 3.8, ON: 4.8 ± 3.4; Ca_V_1.4-IT: OFF: 1.04 ± 0.7, ON: 0.8 ± 0.8; photopic: WT OFF: 81.9 ± 2.4, ON: none; Ca_V_1.4-IT: OFF: 3.9 ± 2.7, ON: none. WT: N = 4; Ca_V_1.4-IT: N = 7; mean ± SEM. Statistics: *** *p* < 0.0001, unpaired t test. All data are presented as mean ± SEM.

Since we detected release from cones, we investigated the dysfunction of pathways involving the cone-to cone bipolar cell synapse. We addressed this hypothesis of a cone-to-cone bipolar cell transmission failure by pharmacologically isolating the OFF cone bipolar cell pathway as this is feasible without blocking synaptic transmission of other glutamatergic synapses in inner retina. We made use of L-AP4, a group III mGluR agonist which binds to mGluR6 receptors expressed in depolarizing ON-bipolar cells, thereby eliminating rod- and cone-driven ON responses in ganglion cells as well as rod-driven OFF responses. The cone-to-OFF cone bipolar cell pathway (Supplementary Fig. 2), however, remained available and OFF ganglion cells would respond to a dark flash provided that OFF cone bipolar cells received input. As expected, the application of 50 µM L-AP4 abolished all ON responses in wild type (Fig. 4a,d) and Ca_V_1.4-IT retinas (Fig. 4d). Wild type OFF responses were still present under both scotopic (with reduced strength due to the block of the primary rod pathway) and photopic conditions (Fig. 4a–d). By contrast, Ca_V_1.4-IT retinas exhibited only baseline ganglion cell activity and light-induced OFF responses were strongly reduced (Fig. 4b,c). In Ca_V_1.4-IT, merely 1% of the OFF ganglion cells kept responding during L-AP4 perfusion under scotopic condition (compared to 76.9% in wild type) and 3.9% were L-AP4 resistant under photopic illumination (vs. 81.9% in wild type) (Fig. 4d). These data indicated a defect in the secondary rod and in the cone pathway of Ca_V_1.4-IT retinas both involving the cone-to-OFF cone bipolar cell synapse.

Because group III mGluRs are also expressed in other retinal neurons^41^, we did further experiments in which we perfused the whole-mounts with 2 µM strychnine. As a competitive antagonist of glycine-gated Cl^−^ channels^42^, strychnine (yellow in Supplementary Fig. 2) blocks the input to OFF cone bipolar cells by the AII amacrine cell, hence affects ganglion cell OFF responses driven via the primary rod pathway. The secondary and tertiary (rod → (cone →) OFF cone bipolar cell) rod pathways would not be affected by this manipulation. We expected similar defects in the OFF pathway under scotopic conditions as seen under L-AP4 perfusion in Ca_V_1.4-IT retinas if the secondary rod pathway is indeed dysfunctional.

In fact, only 28.3% (scotopic) and 7.2% (photopic) of the Ca_V_1.4-IT OFF ganglion cells continued responding during 2 µM strychnine perfusion compared to 81.2% (scotopic) and 87.9% (photopic) of wild type still showing an OFF response (Supplementary Fig. 3a–d). The strychnine-resistant Ca_V_1.4-IT OFF ganglion cells showed a “delayed” light-induced response, a behaviour that was never observed in wild type retinas. These “delayed” responses accounted for all responding Ca_V_1.4-IT OFF ganglion cells observed during strychnine perfusion. We concluded that the scotopic and photopic OFF responses in Ca_V_1.4 IT ganglion cells were driven by the primary rod pathway. As photopic ON responses in Ca_V_1.4-IT ganglion cells were even less preserved than OFF responses it seems likely that also transmission to ON cone bipolar cells is similarly affected, even though we could not test this directly. Together these findings supported the notion of a cone-to-cone bipolar cell transmission defect, thus we analysed the morphology of different second-order neurons to elucidate the effect on the postsynaptic side.

### Morphology of cone bipolar and horizontal cells neurons in Ca_V_1.4-IT retinas

**Figure 5:**
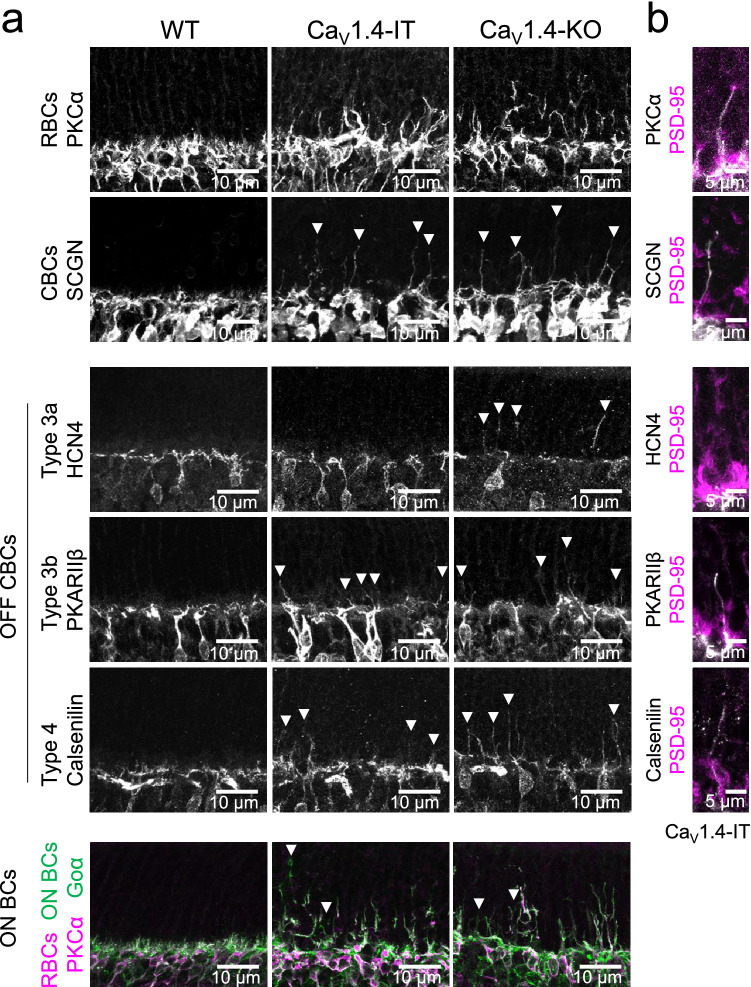
Dendritic morphology of bipolar cells in wild type, Ca_V_1.4-IT and Ca_V_1.4-KO mouse retinas. (**a**) From top to bottom: rod bipolar cells: protein kinase C α (PKCα), type 2–6, 8 cone bipolar cells: secretagogin (SCGN), type 3a cone bipolar cells: hyperpolarization-activated cyclic nucleotide-gated channel 4 (HCN4), type 3b cone bipolar cells: protein kinase A regulatory subunit Iiβ (PKARIIβ) and type 4 cone bipolar cells: calsenilin. A co-staining for PKCα (magenta) together with anti-G protein Goα (green, all ON bipolar cells) distinguished rod bipolar cells from ON-cone bipolar cells. (**b**) Top, a rod bipolar cell sprout contacting a mislocated spherule in the outer nuclear layer. We did not find any evidence that cone bipolar cells approached displaced rods. WT: N = 5–6; Ca_V_1.4-IT: N = 5–6; Ca_V_1.4- KO: N = 3–4.

Dendritic sprouting of postsynaptic cells as a hallmark of outer retina synaptic dysfunction was evident in rod (RBCs labelled with PKCα, Fig. 5a) and cone bipolar cells (CBCs labelled with secretagogin, SCGN; labelling OFF types 2, 3, 4, 5 and ON types 6 and 8) of Ca_V_1.4-IT retinas. We examined dendritic sprouting more specifically in both OFF cone (types 3a, 3b and type 4 stained by HCN4, PKARIIβ and calsenilin, respectively) and ON bipolar cells (Goα) (Fig. 5a). Since anti-Goα labels all ON-type bipolar cells, we identified ON cone bipolar cell processes by co-staining rod bipolar cell dendrites with PKCα. We compared Ca_V_1.4-IT with wild type and Ca_V_1.4-KO retinas, using the same markers. While secretagogin, PKARIIβ, calsenilin and Go_α_ stainings were similar in Ca_V_1.4-KO and Ca_V_1.4-IT, the HCN4 staining showed no sprouting of type 3a OFF cone bipolar cells in Ca_V_1.4-IT retinas. The elongated dendrites of cone bipolar cells did not contact mislocated rod terminals in the ONL (rod terminals labelled with PSD-95, Fig. 5b), in contrast 72.5 ± 7.1% of the rod bipolar cell sprouts contacted a spherule (Ca_V_1.4-IT: N = 3, n = 126). Conversely, 51.5 ± 5.2% of displaced rod spherules were contacted by a rod bipolar cell sprout (N = 3, n = 179, example Fig. 5b, top).

**Figure 6:**
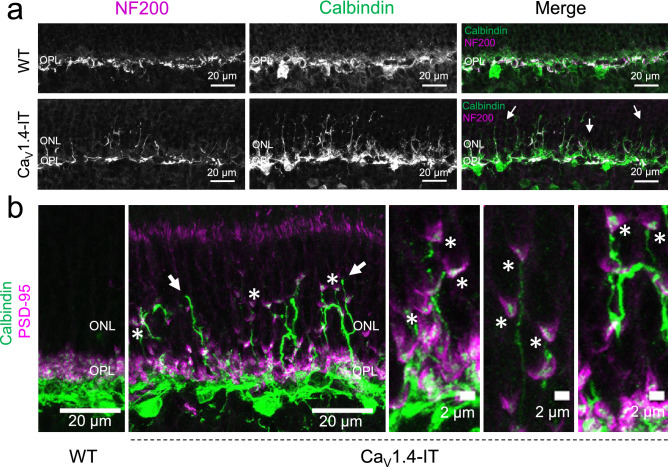
Axonal sprouting of horizontal cells in Ca_V_1.4-IT retinas. (**a**) Double-labelling of horizontal cell processes with neurofilament 200 (NF200) and calbindin in wild type and Ca_V_1.4-IT retinas. The white labelling in the merge shows that in Ca_V_1.4-IT retinas Calbindin-labelled processes (green) were mostly overlapped with the NF200 (magenta) labelling indicating an axonal origin of the majority of sprouting horizontal cell processes. Arrows indicate dendritic sprouts of horizontal cells. WT: N = 4, Ca_V_1.4-IT: N = 4. (**b**) The sprouting processes approached displaced rod spherules labelled with PSD-95 in the ONL (marked by *) with few exceptions (marked by arrows). WT: N = 4, Ca_V_1.4-IT: N = 5.

Like type 3a cone bipolar cell dendrites, also horizontal cell dendrites were maintained in the OPL presumably connecting to cone terminals^43^, while their axonal arbors rewired with displaced rod terminals. We showed this using Neurofilament 200 (NF200), a marker for horizontal cell axons^44,45^, co-labelling all horizontal cell processes with Calbindin to distinguish dendritic (calbindin-only) from axonal processes (NF200 and calbindin double-labelled). In Ca_V_1.4-IT retinas, most sprouting processes were co-labelled with both markers and therefore likely of axonal origin (Fig. 6a) with few exceptions (Fig. 6a, arrows), indicating that horizontal cell dendritic sprouting was rare.

Furthermore, our analyses showed that the majority of Ca_V_1.4-IT horizontal cell sprouts contacted displaced rod spherules, and that most displaced spherules were approached by horizontal cell processes (Fig. 6b, asterisk), with few exceptions (Fig. 6b, arrows). These findings are consistent with a preservation of horizontal cell-cone contacts.

### Modulation of ganglion cell responses by dihydropyridines

While an increased intracellular Ca^2+^ concentration was proposed at the ribbon synapse, reported in Ca^2+^ imaging experiments^23^ mainly from rod terminals (in line with the pronounced hyperpolarizing shift seen in the voltage-dependence of Ca_V_1.4-IT channels^29^) cone photoreceptors retained the ability of releasing glutamate (Fig. 3). However, the cone-to-cone bipolar cell synapses were not transmitting modulations of light intensity (as in our flash stimuli) efficiently, revealed by the cone and secondary rod pathway dysfunction proven in our L-AP4 and strychnine experiments. We hypothesized that, if an increased baseline Ca^2+^ level in photoreceptor terminals was mainly responsible for the dysfunction we observed (response delays and failures to respond), then we should be able to reverse some of the effects by reducing the basal Ca^2+^ load. Hence, we tested whether we could ameliorate the ganglion cell phenotype, using the response delay as a robust read out parameter, by blocking retinal LTCCs in Ca_V_1.4-IT retinas. We had to conduct these experiments in scotopic light level because only there did we get enough responding ganglion cells for a meaningful analysis, thus involving a potential rescue of the secondary rod pathway in addition to potential improvements on rod release as a proxy for cone terminal function.

**Figure 7:**
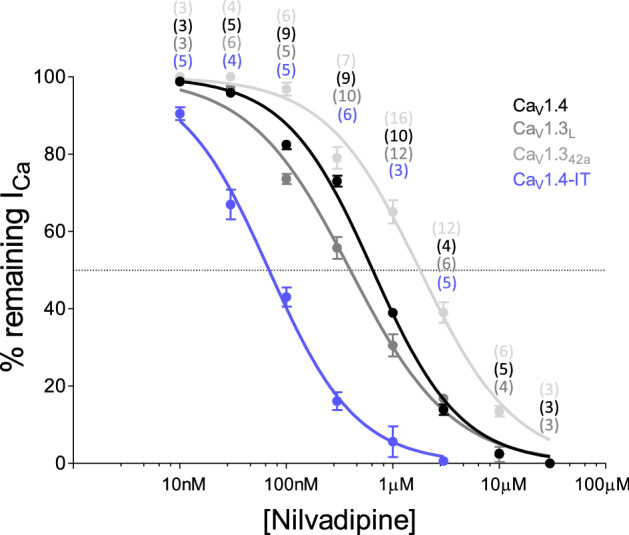
Concentration–response curves for calcium current inhibition by nilvadipine. In the nilvadipine dose–response curve the percentage of the remaining calcium current in the presence of different nilvadipine concentrations is indicated for human Ca_V_1.4-WT (black), Ca_V_1.4-IT (blue), Ca_V_1.3_L_ (dark grey) and Ca_V_1.3_42a_ (light grey) channels. The dotted line indicates how the IC_50_ was determined. All data points represent mean ± SEM; the number of cells recorded is indicated in the graphs.

The dihydropyridine LTCC blocker nilvadipine has previously been reported in different studies to decrease LTCC mediated currents in photoreceptors^46–49^ but the effective concentration to block Ca_V_1.4 channels was unknown. Therefore we first determined the nilvadipine sensitivity of Ca_V_1.4 and Ca_V_1.4-IT channels in a heterologous expression system. Remarkably, the Ca_V_1.4-IT mutation increased the nilvadipine sensitivity almost tenfold (Fig. 7). In a second step, we evaluated the inhibition of two Ca_V_1.3 splice isoforms^50^ by nilvadipine: full-length Ca_V_1.3 (Ca_V_1.3_L_) and a variant with shorter C-terminus (Ca_V_1.3_42a_). Nilvadipine blocked the calcium channels with different IC_50_ with the highest affinity for Ca_V_1.4-IT (Supplementary table 3; Fig. 7: Ca_V_1.4-IT < Ca_V_1.3_L_ < Ca_V_1.4 < Ca_V_1.3_42a_).

The prerequisite for further functional analyses in the retina was, that the Ca_V_1.4-IT ganglion cells were still able to respond at scotopic dark (OFF) flashes (Fig. 4b, control condition), suggesting that not all calcium channels were open at background light levels. Thus, further depolarization induced by the negative contrast could indeed trigger an additional light-dependent glutamate release.

**Figure 8:**
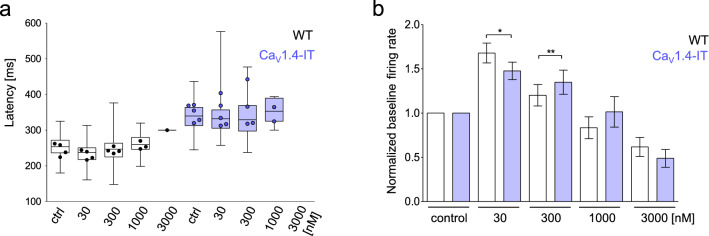
Effect of the L-type calcium channel blocker nilvadipine on retinal ganglion cell activity. (**a**) ON and OFF ganglion cell light response latencies measured under scotopic conditions; in the box plot data are mean ± SD given in [ms], n describes the number of responding ganglion cells: wild type (WT): control ringer solution: 255.1 ± 36.7, n = 156; nilvadipine: 30 nM: 239.1 ± 33.6, n = 158; 300 nM: 256.6 ± 34.9, n = 151; 1 µM: 271.6 ± 35.1, n = 103; 3 µM: 300.1 ± 0, n = 1. Ca_V_1.4-IT: control ringer solution: 339 ± 43.6, n = 69; nilvadipine: 30 nM: 339.3 ± 64.15, n = 61, 300 nM: 336 ± 61.9, n = 42, 1 µM: 355.0 ± 30.9, n = 11, 3 µM: none responding. Box and whiskers datasets represent mean, 25th to 75th percentiles and minimum to maximum values for all the ganglion cells recorded. The circles, however, represent the number of animals with responding ganglion cells: WT, N = 4; Ca_V_1.4-IT, N = 4. No statistical difference was observed. (**b**) The baseline firing activity was recorded 5 s after the full-field flash under grey background stimulation. For each concentration, the baseline firing rate was normalized to the control condition: data are presented as mean ± SEM in [Hz], WT: nilvadipine: 30 nM: 1.67 ± 0.11; 300 nM: 1.20 ± 0.12; 1 µM: 0.83 ± 0.12; 3 µM: 0.61 ± 0.11, n = 158; IT: nilvadipine: 30 nM: 1.48 ± 0.10; 300 nM: 1.35 ± 0.14; 1 µM: 1.01 ± 0.17; 3 µM: 0.49 ± 0.10, n = 69. WT, N = 4. Ca_V_1.4-IT, N = 4. Two-tailed Mann Whitney test, *, *p* = 0.0195; **, *p* = 0.0013.

We then recorded wild type and Ca_V_1.4-IT ganglion cell responses at scotopic light level in the presence of different concentrations of nilvadipine (30 nM to 3 µM; Fig. 8). In wild type, we also expected a decrease in the ganglion cell response latency upon blocking LTCCs, at least with some concentrations, because a reduction of Ca^2+^ influx would mimic a more hyperpolarized state of the photoreceptors, akin to light adaptation. Indeed, in the presence of 30 nM nilvadipine a trend towards a faster response was observed compared to the control ringer solution (Fig. 8a). But we found no statistically significant differences in any concentration compared to control. In Ca_V_1.4-IT retinas, other than an increased variability, we did not observe any change in the light-induced ganglion cell response delay compared to control.

However, the ganglion cell baseline firing frequency, measured during constant mean background grey levels, increased upon nilvadipine perfusion compared to control conditions in both wild type and mutant retinas. The baseline frequencies decreased again with increasing nilvadipine concentrations, always in a comparable fashion in Ca_V_1.4-IT and wild type retinas, and at 3 μM were lower than in the control condition (Fig. 8b). Of note, when we perfused wild type retinas with 1 μM nilvadipine more than 60% of the ganglion cells showed light responses, while only in two out of four Ca_V_1.4-IT retinas a few cells were still responding. During 3 µM nilvadipine perfusion only one wild type retina was still active (Fig. 8a), while Ca_V_1.4-IT retinas showed no responses. These data fit with the nilvadipine sensitivity for wild type and mutant Ca_V_1.4-IT channels seen in heterologous expression systems. In conclusion, a reduction of basal Ca^2+^ influx by a dihydropyridine LTCC blocker is not sufficient to rescue deficits in photoreceptor synaptic transmission caused by the Ca_V_1.4-IT mutation.

## Discussion

The variability of symptoms in human CSNB2 patients ranges from night blindness to light sensitivity indicating changes in both rod and cone pathways. The majority of literature, however, focused on rod pathway components; only few abnormalities have been reported in neurons downstream of cones^25,31^. Our results suggested that the Ca_V_1.4-IT mutation does not prevent neurotransmitter release from photoreceptors, but rather induces morphological rearrangement of the retinal network resulting in a dysfunction of the cone pathway. We reported sprouting of ON and OFF cone bipolar cells dendrites that did not contact any displaced rod photoreceptor terminals. This finding is in contrast to rod bipolar cells which have been shown to make ectopic synapses^4,18,19,21,23,25,26^. Rod retraction seems to be a trigger for rod bipolar cell elongation, however, the trigger for cone bipolar cell sprouting is elusive. Of note, type 3a OFF cone bipolar cells were the only subtype not showing dendritic elongation. The underlying reason for this cellular subtype specificity is yet unresolved. A key difference might be found in the receptor composition, as type 3a and type 2 OFF bipolar cells (not investigated here) express only kainate-type glutamate receptors^51^ that induce a longer desensitized state compared to AMPA-type glutamate receptors expressed in other OFF bipolar cell types^52^. However, we showed that cone-contacting horizontal cell dendrites, which largely express AMPA-type glutamate receptors^53^, showed little sprouting and even maintained functional contacts with cones. Therefore different mechanisms might underlie the resilience of type 3a cone bipolar cells and horizontal cells towards perturbances at cone terminals and the ensuing remodelling of the cone contacts.

Although Ca_V_1.4 channels are expressed in both cones and rods^1–4^, the gain-of-function mutation affected cones in a different way than rods. While rods retract their axonal terminals into the ONL and show cell death^4,21,23^, we did not find evidence for cell death in cones. A possible explanation might be related to the photoreceptor specific internal Ca^2+^ modulation. Cone pedicles possess the machinery to remove intra-terminal free Ca^2+^ more rapidly during light adaptation compared to rods spherules^54^. Therefore cones might also be less susceptible to Ca^2+^-induced toxicity. Phenotypic differences in rod and cone phenotype have been found in different mouse models carrying mutations in the Ca_V_1.4 channel complex. While some found that cones are spared^13,17,31,55,56^, other reported drastic changes compared to rods^14,27^. Still, a recent study showed that the Ca_V_1.4.IT mutation can exert different functional phenotypes depending on splice variant and subunit composition^57^. Deeper knowledge about the channel composition in rods and cones will therefore be essential to elucidate the CSNB2 phenotype.

Although in Ca_V_1.4-IT retinas cone terminals remained in the OPL the characteristic mosaic of cone terminals was lost and clusters of smaller pedicles, often branching from the same axon appeared. Cone axonal branching might be related to changes in the Ca^2+^ level in the presynaptic terminal because this phenotype has been observed in two different Ca_V_1.4 KO models^14,25^ and one carrying a Ca_V_1.4 mutation (^21^, this study). It might even be a common feature that cone pedicles cluster while they are still releasing but have lost most of their downstream partners as suggested previously also for horizontal cell ablated mouse retinas^58^. Nevertheless, we would have expected a more pronounced difference in wild type and Ca_V_1.4-IT EPSCs. Still, our horizontal cell recordings were performed on under ambient room light conditions and we cannot exclude a more pronounced defect on light-induced vesicle release not revealed during steady-state release. While the regulation of vesicle release would be an important question to address, we cannot exclude that the strong impairment in the photopic response is also due to the postsynaptic receptors inability to respond to the changes (leading to e.g. cone bipolar cell sprouting).

Under the hypothesis of a higher Ca^2+^ influx in photoreceptor terminals, the remaining rod-driven signal seen in our MEA experiments allowed us to test whether we can modulate the Ca_V_1.4-IT-mediated Ca^2+^ influx pharmacologically. Yet, we did not see a faster signal transmission in scotopic light as a consequence of the nilvadipine-induced reduction of Ca^2+^ influx. This finding that does not support the notion of a similarly increased intracellular Ca^2+^ load in cone terminals at steady state or a consequentially higher glutamate level in the synaptic cleft which might lead to postsynaptic glutamate receptor desensitisation. Therefore, experiments with a Ca_V_1.4 channel gating modulator would be required to optimize activation and inactivation properties of the mutated channels and thus extend the dynamic range of Ca^2+^ signalling in Ca_V_1.4-IT retinal neurons.

Nevertheless, in the adult mutant retina, molecular and/or structural rearrangements of the inner plexiform layer might contribute to the transmission delay. In fact, in the presence of strychnine, we observed a “delayed” ganglion cell response also in the Ca_V_1.4-IT retina. Further investigations would have exceeded the scope of the present study, but the phenotype was reminiscent of a complex, non-physiological, amacrine cell influence^59^.

We can exclude that our approach failed due to a lack of nilvaldipine block of Ca_V_1.4-IT channels because the mutant channels even showed a tenfold higher sensitivity compared to wild type Ca_V_1.4 channels in a heterologous expression system. Such behaviour was previously shown also in gain-of-function variants of Ca_V_1.3 L-type calcium channels^60,61^ possibly explained by the state-dependent action of dihydropyridines^50,62^. Ca_V_1.3 which has been suggested to be the second major L-type calcium channel in photoreceptor terminals^30^ might have been unaffected by the treatment based on the lower nilvadipine sensitivity that we observed. Still, other L-type calcium channel expressed in the IPL^43,45,63^ might account for the increased baseline firing rate in ganglion cells that we observed, elicited by disinhibition effects (e.g. Ca_V_1.2 has a markedly higher dihydropyridine sensitivity^50,64–66^).

Altogether our data indicated that the rod pathway is still active whereas pathways involving cone-to-cone bipolar cell transmission including the secondary pathway, are strongly impaired. In human CSNB2 patients that show a congenital rod-cone dysfunction^8^ this difference might also be reflected and should be considered in the ERG interpretation (e.g. influence of secondary rod pathway in the scotopic ERG^67,68^) and when treatment options are discussed. The fact that the gain of channel function in adult Ca_V_1.4-IT mice cannot be reversed by simply decreasing the Ca^2+^ levels will have to be taken into account for pharmaco-therapeutic approaches which aim at restoring channel function. It might nevertheless prove beneficial to use low-dose Ca^2+^ channel blockers for long term treatments aimed to reduce Ca^2+^ overload and toxicity in the retina, as we’ve seen only moderate acute effects on ganglion cells responses.

## Supplementary Information

Supplementary Information 1.

## Data Availability

All data generated or analyzed during this study are included in this published article (and its Supplementary Information files).
