# Supplementary material for: Function of cone and cone-related pathways in CaV1.4 IT mice
Source: Sci Rep. 2021 Feb 1;11:2732. doi: 10.1038/s41598-021-82210-7 (PMC7851161; doi:10.1038/s41598-021-82210-7)
Supplement: Supplementary file 1 — Supplementary Information 1. [file 41598_2021_82210_MOESM1_ESM.pdf]

Supplementary information

Manuscript title: Function of cone and cone-related pathways in Cav1.4 IT mice.

Authors:

Lucia Zanetti[1], Irem Kilicarslan[1], Michael Netzer[1], Norbert Babai[2], Hartwig Seitter[1] and Alexandra Koschak[1*]

[1] University of Innsbruck, Institute of Pharmacy, Pharmacology and Toxicology, 6020 Innsbruck, Austria

[2] University of Erlangen, Department of Biology, 91058 Erlangen, Germany

* Correspondence to Alexandra Koschak, University of Innsbruck, Institute of Pharmacy, Pharmacology and Toxicology, Center for Chemistry and Biomedicine, Innrain 80-82, A-6020 Innsbruck Austria, phone: +43-(0)512-507-58807, Fax: +43-(0)512-507-58899, email: alexandra.koschak@uibk.ac.at.

Supplementary methods

Cell line and cell culture

Culture medium DMEM (Catalogue # D6546, Sigma-Aldrich) supplemented with 10% fetal bovine serum (Catalogue # 10270-106, Invitrogen), 2 mm l-glutamine (Catalogue # 25030-032, Invitrogen), 10 U/ml penicillin G (Catalogue # P3032, Sigma-Aldrich), and 10 U/ml streptomycin (Catalogue # S6501, Sigma-Aldrich), was use for culturing. Cells were grown at 37°C in a humidified incubator with 5% CO_2 and split when they reached ~80% of confluence using 0.05% trypsin. $Ca_V1.4$ and $Ca_V1.4$-IT α1 subunits were transiently transfected using the Ca^{2+}-phosphate precipitation method together with eGFP as transfection marker in HEK-293 cell stably expressing β3 and α2δ1 [1]. Cells were plated onto poly-l-lysine-precoated 35 mm culture dishes and used for experiments 20-72h after transfection. For patch-clamp experiments in stable cell line expressing $Ca_V1.3_L$ and $Ca_V1.3_{42a}$ together with β3 and α2δ1 subunits, cells were directly plated onto poly-l-lysine-precoated 35 mm culture dishes, the expression of α1-subunit was induced using 1 µg/ml doxycycline (Catalogue # D1822, Sigma-Aldrich), kept <5% CO2 at 30°C and used 20–72 h after induction. For maintenance of stable cell lines, selection agents for each subunit were applied every 3 weeks for 5 days [α1, 50 µg/ml hygromycin B; β3, 500 µg/ml geneticin (Catalogue # 10131–027, Invitrogen); and α2δ-1, 15 µg/ml blasticidin S (Catalogue # A11139–03, Invitrogen)].

Whole-cell patch-clamp recordings in HEK293 cells

All electrophysiological experiments were carried out at room temperature. For whole-cell patch-clamp recordings, electrodes with a resistance of 1.5–3.5 MΩ were pulled from glass capillaries (borosilicate glass; Catalogue # 64-0792, Harvard Apparatus) using a micropipette puller (Sutter Instruments) and fire polished with an MF-830 Microforge (Narishige). The intracellular solution contained (in mmol/L): 135 CsCl, 10 Cs-EGTA, 1 $MgCl_2$, 10 HEPES, and 4 ATP-Na2 adjusted to pH 7.4 with CsOH. The bath solution contained (in

mmol/L): 15 $CaCl_2$, 150 choline-Cl, 1 $MgCl_2$, and 10 HEPES, adjusted to pH 7.3 with CsOH. Cells were perfused with bath solution at a flow rate of 0.5 ml/min in absence or presence of different concentrations of nilvadipine (Catalogue #5711, Tocris Bioscience). The perfusion needle of the focal air-pressure driven system (OctaFlow, ALA Scientific Instruments) was placed in a fixed position in proximity of the patching pipette. Control recordings with only vehicle were performed on each day before any drug perfusion experiment to exclude drug contamination. Cells were recorded in the whole-cell patch-clamp configuration using an Axopatch 200B Amplifier (Molecular Devices). Recordings were digitized (Digidata 1322A Digitizer, Molecular Devices) at 50 kHz, low-pass filtered at 2 kHz, and subsequently analysed using pClamp 10.2 software (Molecular Devices) and custom made Matlab script Matlab (The Mathworks Inc., MA, USA). Compensation was applied for 60–90% of the series resistance. For the characterization of the voltage dependence of the stable cell lines, currents of <200 or >3500 pA were excluded. All voltages were corrected for a liquid junction potential of −9 mV offline. Current–voltage (I–V) relationships were obtained by applying a 50-ms square pulse to various test potentials starting from a holding potential (HP) of −89 mV. I–V curves were fitted to the following equation: I = Gmax(V – Vrev)/(1 + exp[(V0.5 – V)/k] where I is the peak current amplitude, Gmax is the maximum conductance, V is the test potential, Vrev is the extrapolated reversal potential, V0.5 is the half-maximal activation voltage, and k is the slope factor. The activation threshold (V_{tresh}) was defined as the voltage reached 5% of the maximal current.

Supplementary figures

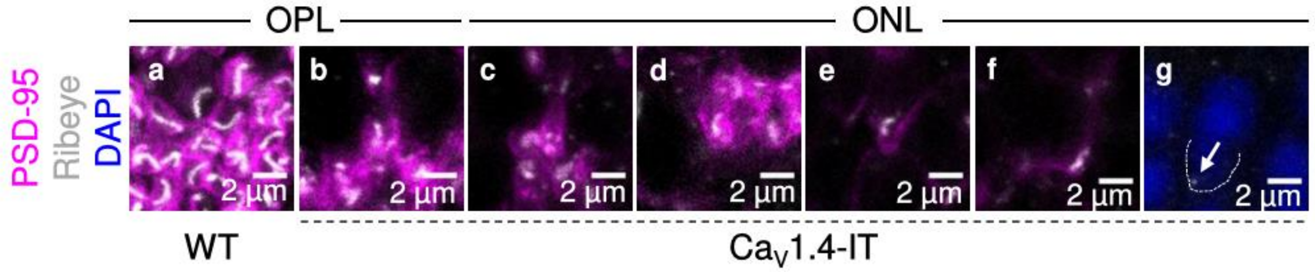

Supplementary figure 1: Shape of synaptic ribbons during retraction. In wild type (WT) retinas **(a)**, rod spherules (PSD-95) were located only in the outer plexiform layer (OPL) containing ribbons with horseshoe-like appearance (Ribeye). In Ca$_V$1.4-IT retinas rod terminals retracted into the outer nuclear layer (ONL). The shape of the synaptic ribbons changed from elongated **(b-f**) to circular **(g)** at the end of the retraction process. The arrow in (g) points to a representative ribbon that showed a circular shape once the rod spherule reached perinuclear position. PSD-95 was omitted for better clarity and is represented by the hand-drawn line. The photoreceptor nuclei were stained with DAPI (blue). WT: N = 6; Ca$_V$1.4-IT: N = 6.

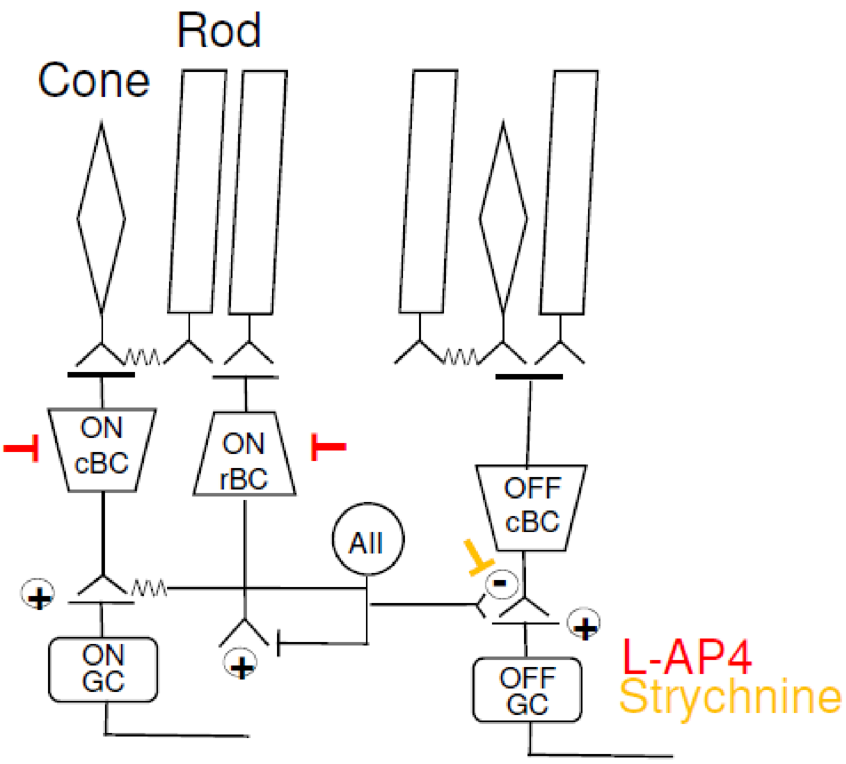

Supplementary figure 2. Schematic diagram of rod and cone signalling pathways. ON pathway: ON rod bipolar cells (ON rBC; middle) receive input from rods and make excitatory glutamatergic synapses onto AII amacrine cells (AII) which in turn form electrical synapses with ON cone bipolar cells (ON cBC) (primary rod pathway). The ON cBCs then synapse to ON ganglion cells (ON GC). ON cBCs (left) receive input from cones. OFF pathway: OFF cBCs (right) receive input from cones which form synapses with OFF ganglion cells (OFF GC). AII cells which receive input from rBCs form inhibitory glycinergic synapses with OFF cBC. Electrical synapses are formed between rods and cones; the latter carry rod signals also to ON cBCs but also OFF cBCs (secondary rod pathway). The tertiary rod pathway is omitted for better clarity. Other abbreviations and symbols: +, sign preserving synapse; -, sign converting synapse; zigzags; electrical synapses; L-AP-4, L-2-amino-4-phosphonobutyrric acid; strych, strychnine.

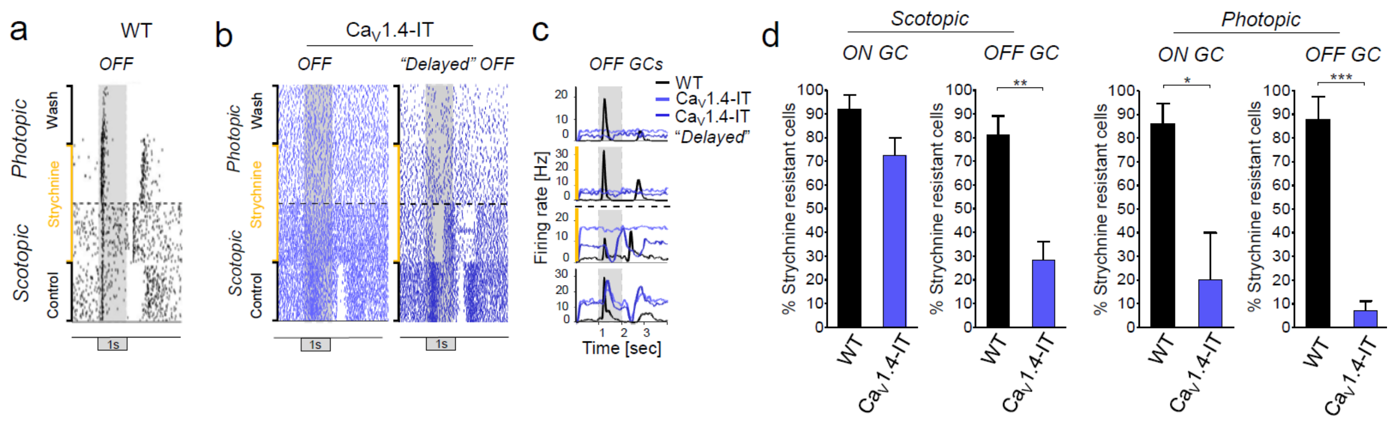

Supplementary figure 3. Strychnine perfusion in WT and Cav1.4-IT retinas.

OFF signalling was isolated by using the glycinergic antagonist strychnine (2 µM, yellow axis). In (a) and (b), representative example on OFF WT and Cav1.4-IT ganglion cell spiking activity upon negative contrast flash (light grey) light stimulation under scotopic and photopic conditions are indicated. 40 repetitions of the same stimulus are depicted. (**a**) In WT, strychnine only abolishes the direct component of the OFF scotopic pathway, leaving available the secondary rod pathway. In Cav1.4-IT, the majority of OFF GC did not respond any light stimulation (b, left panel) or with a "delayed" response (b, right). (c) shows the peri-stimulus histogram of the sum of data in (a) and (b). (d) Percentage of ganglion cells (wild type, WT, black and Cav1.4-IT; blue) responding to different light illumination under strychnine perfusion (% strychnine resistant cells): scotopic: WT: OFF: 81.2 ± 15.8, ON: 91.96 ± 11.8; Cav1.4-IT: OFF: 28.3 ± 17.3, ON: 79.3 ± 17.4; photopic: WT: OFF: 87.9 ± 19.02, ON: 86.07 ± 17.3; Cav1.4-IT: OFF: 7.2 ± 6.9, ON: 20.0 ± 28.3. WT: N = 4; Cav1.4-IT: N = 5; mean ± SEM. The number of responding ganglion cells prior to drug perfusion was set to 100% (WT, ON n = 46; OFF n = 38; Cav1.4-IT, ON n = 22; OFF n = 89). Statistics: * $p < 0.05$, ** $p < 0.01$, *** $p < 0.001$, unpaired t test.

Supplementary tables

Supplementary table 1: List of Primary Antibodies

Protein	Species	Working dilution	Company, catalogue number
Cone arrestin	Rabbit	1:1000	Sigma Aldrich, AB15282
PSD-95	Rabbit	1:1000	Synaptic Systems, 124 002
PSD-95	Mouse	1:1000	Thermo Scientific MA1-045
Ribeye	Rabbit	1:500	Synaptic Systems, 192 103
Secretagogin	Sheep	1:1000	BioVendor, RD184120100
HCN4	Rabbit	1:250	Alomone Labs, APC-052
PKARIIβ	Mouse	1:2000	BD Biosciences, 610626
Calsenilin	Mouse	1:2000	Millipore, 05-756
PKCα	Mouse	1:200	Santa Cruz, Sc-8393
PKCα	Rabbit	1:200	Santa Cruz, Sc-208
Go_{α}	Mouse	1:500	Sigma Aldrich, MAB3073
RBPMS	Guinea-pig	1:500	PhosphoSolutions, 1832
Calbindin	Rabbit	1:1000	Swant CB-38a
NF200	Chicken	1:2000	Abcam ab4680

Supplementary table 2: List of Secondary Antibodies

Antibody	Working dilution	Company, catalogue number
Alexa Fluor® 488 donkey-anti-rabbit IgG (H+L)	1:400	Invitrogen, A-21206
Alexa Fluor® 488 goat-anti-mouse IgG (H+L)	1:400	Invitrogen, A-11001
Alexa Fluor® 488 goat-anti-chicken IgG (H+L)	1:400	Invitrogen A-11039
Alexa Fluor® 568 goat-anti-mouse IgG (H+L)	1:400	Invitrogen, A-11004
Alexa Fluor® 568 goat-anti-rabbit IgG (H+L)	1:400	Invitrogen, A-11011
Alexa Fluor® 568 goat-anti-guinea pig IgG (H+L)	1:400	Invitrogen, A-11075
Alexa Fluor® 568 donkey-anti-sheep IgG (H+L)	1:500	Abcam, Ab175712

Supplementary table 3: Nilvadipine IC_{50} values for the inhibition of different L-type calcium channel isoforms and mutants. IC_{50}, half maximal inhibitory concentration; $LogIC_{50}$, logarithmic value of the IC_{50}, R2, coefficient of determination of the goodness of the fit; Slope, or Hill-Slope, steepness of the curve. Data are shown as mean ± SEM

	**IC_{50} (95% CI) [nM]**	**$LogIC_{50}$ [M]**	**R^2**	**Slope**
$Ca_V1.4$	658.9 (586.3 – 740.5)	-6.18 ± 0.05	0.9831	1.06 ± 0.12
$Ca_V1.4$-IT	69.4 (60.7 – 79.3)	-7.16 ± 0.06	0.9735	1.05 ± 0.13
$Ca_V1.3_L$	399.2 (349.1 – 456.5)	-6.40 ± 0.03	0.9591	0.91 ± 0.06
$Ca_V1.3_{42a}$	1813.0 (1573.9 – 2103.7)	-5.74 ± 0.03	0.9214	0.96 ± 0.08

Reference

1 Ortner, N. J. *et al.* Lower Affinity of Isradipine for L-Type Ca(2+) Channels during Substantia Nigra Dopamine Neuron-Like Activity: Implications for Neuroprotection in Parkinson's Disease. *J Neurosci* **37**, 6761-6777, doi:10.1523/JNEUROSCI.2946-16.2017 (2017).
